# Neuropeptide Substance-P-Conjugated Chitosan Nanofibers as an Active Modulator of Stem Cell Recruiting

**DOI:** 10.3390/ijms17010068

**Published:** 2016-01-07

**Authors:** Min Sup Kim, Sang Jun Park, Wheemoon Cho, Bon Kang Gu, Chun-Ho Kim

**Affiliations:** Laboratory of Tissue Engineering, Korea Institute of Radiological and Medical Science, Seoul 139240, Korea; kimins21@kirams.re.kr (M.S.K.); sjpark@kirams.re.kr (S.J.P.); whgnlans@hanmail.net (W.C.); bons1020@kirams.re.kr (B.K.G.)

**Keywords:** chitosan, substance-P, wound healing, electrospinning, human mesenchymal stem cell, tissue engineering

## Abstract

The goal to successful wound healing is essentially to immobilize and recruit appropriate numbers of host stem or progenitor cells to the wound area. In this study, we developed a chitosan nanofiber-immobilized neuropeptide substance-P (SP), which mediates stem cell mobilization and migration, onto the surfaces of nanofibers using a peptide-coupling agent, and evaluated its biological effects on stem cells. The amount of immobilized SP on chitosan nanofibers was modulated over the range of 5.89 ± 3.27 to 75.29 ± 24.31 ng when reacted with 10 to 500 ng SP. *In vitro* migration assays showed that SP-incorporated nanofibers induced more rapid migration of human mesenchymal stem cells on nanofibers compared to pristine samples. Finally, the conjugated SP evoked a minimal foreign body reaction and recruited a larger number of CD29- and CD44-positive stem cells into nanofibers in a mouse subcutaneous pocket model.

## 1. Introduction

Wound healing is the dynamic and complicated process involving extracellular matrix (ECM), signaling molecules, immune and stem cells and many other factors. Because of the pathological and physiological complexity of the wound-healing process, perfect tissue regeneration is difficult to achieve [1,2]. Following skin damage, in particular, various signaling bio-active molecules initiate the processes of stem cell mobilization [3,4]. Signaling bio-active molecules, including interleukin (IL) series (-3, -7, -8 and-12), vascular endothelial growth factor (VEGF) and insulin-like growth factor (IGF), as well as stromal cell-derived factor (SDF)-1α, can induce local stem cell mobilization [5]. For successful wound healing, appropriate numbers of host stem or progenitor cells should be mobilized and recruited to the wound area. However, during the normal wound-healing process, the portions of adult stem cells are not sufficient to play active roles in tissue regeneration [6].

Substance-P (SP) is a known member of the tachykinin neuropeptide released from the central nervous system (CNS), involving angiogenesis, wound-healing and mobilization and proliferation of inflammatory host cells [7,8]. As an active wound-healing modulator, SP is known to induce the recruitment and migration of bone marrow-derived mesenchymal stem cells (MSCs) [9]. Recent studies have shown that SP inspires the recruitment of hematopoietic stem cells, stromal-like CD29-positive cells and MSCs, which can participate in a regeneration process at the injury site [9,10]. However, when soluble SP is injected into the tissue, SP is enzymatically degraded under *in vivo* conditions [11]. Thus, the development of a delivery system is key to us having success in the therapeutic applications of bioactive proteins.

One candidate material for use in delivery vehicles for therapeutic agents is chitosan, a material from sea crustaceans that possesses low immunogenic, biodegradable and biocompatible properties [12,13]. Furthermore, because of their positive-charged amino groups, this functional group confers unique properties that incorporate bio-active molecules, such as cytokines and synthetic peptides [14,15]. For the ultimate delivery system using chitosan, the nanofibrous structures prepared by electrospinning have a high surface area, onto which various bio-active molecules of high density could be immobilized [16]. Previously, we reported the controlled release of SP from chitosan nanofibers (CNs) prepared using a peptide-coupling agent that resulted in enhanced metabolic activities of human mesenchymal stem cells (hMSCs) *in vitro* [17]. Due to their high surface area-to-volume ratio, CNs provided ultimate advantages as active substrates for hMSCs’ proliferation and as big depots for SP delivery.

In this study, we prepared CNs using the electrospinning method and then sequentially incorporated SP on the nanofibers using a peptide-coupling process. To explore the potential for wound dressing applications, we observed the morphological stability on incorporated SP content and investigated the *in vitro* metabolic activity and migration behavior of hMSCs cultured on them. Finally, we evaluated foreign body reactions (FBRs) and MSC recruitment in subcutaneously-inserted, SP-immobilized CNs *in vivo*.

## 2. Results and Discussion

### 2.1. Morphological Analysis of Nanofibers

**Figure 1:**
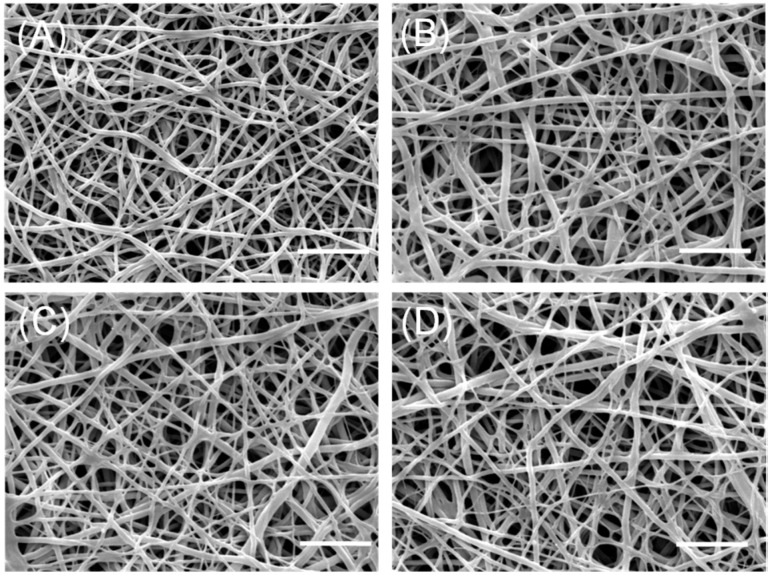
Representative SEM images of pristine chitosan nanofibers (CNs) (**A**); neutralized CNs (**B**); CNs with passively-adsorbed substance-P (SP) (P-CNs) (**C**); and CNs with covalently-immobilized SP (S-CNs) (**D**). Scale bar: 5 μm.

First of all, we observed the morphological stability of nanofibers during the aqueous neutralization and SP coupling process. Scanning electron microscopy (SEM) images of pristine or non-neutralized CNs (Figure 1A) presented a highly uniform and smooth nanofibrous morphology without defects. Then, NaOH treatments for neutralization hold their nanofibrous structure (Figure 1B), consistent with our previous study [18]. Furthermore, this fibrous morphology of nanofibers was maintained during the SP coupling process (Figure 1C,D). From SEM analysis, the aqueous neutralized and immobilized processes do not significantly change the morphology of fibers.

**Figure 2:**
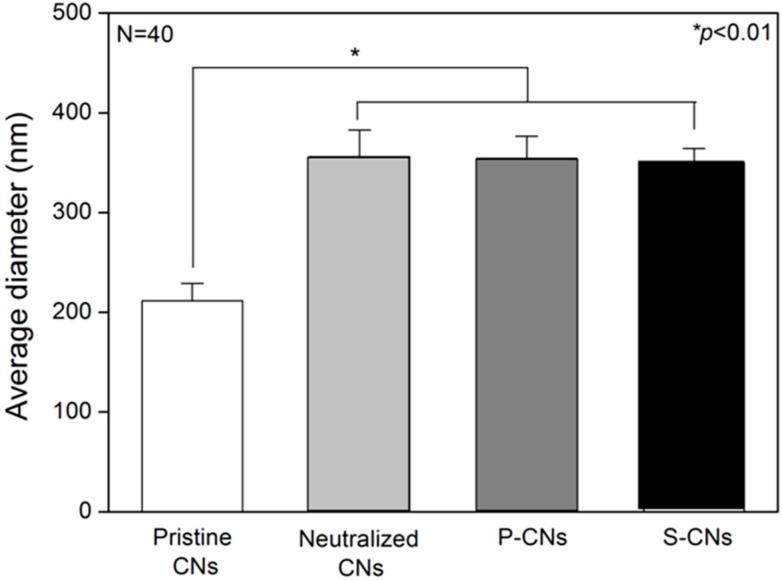
Average fiber diameters of CNs. The average diameter of nanofibers was determined by selecting 10 fields from each image and manually measuring fiber diameters.

However, after completion of the aqueous SP coupling process, the fibers showed a thickened shape (compare to Figure 1A). To analyze these morphological changes quantitatively, we measured the average diameters of CNs from the SEM images of Figure 1. In Figure 2, the average diameter of pristine CNs samples was 211.43 ± 17.44 nm, which increased to 356.22 ± 26.45 nm on the neutralized CNs. This phenomenon could be explained by the presence of minimum water in NaOH solution (dissolved in methanol) causing the swelling or partial hydrolysis of the chitosan component during the neutralization process [18]. The average diameters of passively-adsorbed SP (P-CNs) and covalently-immobilized SP (S-CNs) were 354.27 ± 22.29 and 349.83 ± 14.51 nm, respectively, which were similar to those of neutralized nanofibers.

### 2.2. Quantification of SP on Nanofibers

**Figure 3:**
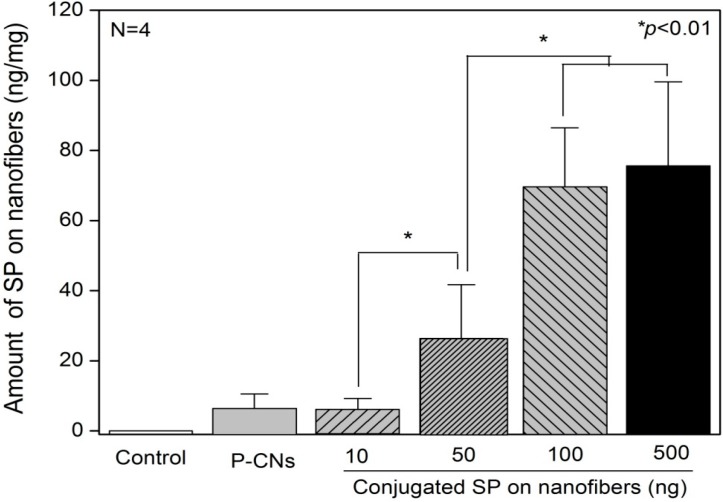
The amount of SP on nanofibers was analyzed using ELISA. SP was passively-adsorbed onto nanofibers (P-CNs) at a single concentration (100 ng) or actively immobilized on nanofibers (S-CNs) at different concentrations (10, 50, 100 and 500 ng).

Next, we quantified the amount of passively-adsorbed or immobilized SP on nanofibers using ELISA. In Figure 3, the amount of SP passively-adsorbed on P-CNs was 6.21 ± 4.32 ng. For S-CNs, the amount of immobilized SP was greatly affected by the concentration of SP. Accordingly, for CNs reacted with 10 ng of SP, the amount of immobilized SP was 5.89 ± 3.27 ng, whereas reactions of CNs with 50 and 100 ng of SP further increased the amount of SP to 26.34 ± 15.37 and 69.63 ± 16.82, respectively. At a higher concentration of SP (500 ng), the amount of immobilized SP (75.29 ± 24.31 ng) was similar to that at 100 ng, which appears to be a saturating concentration for immobilization of SP on CNs in our conditions. In our previous study, the releasing behavior of SP from CNs was evaluated *in vitro* conditions over the course of seven days [17]. Passively-adsorbed P-CNs showed a particularly notable initial burst release, with 81.99% ± 10.61% and 93.72% ± 4.91% of SP being released after 12 and 24 h, respectively, after which, a slight increased release of SP was observed. However, immobilized SP (50 and 100 ng) released slowly during seven days, compared to others.

### 2.3. In Vitro Analysis of Nanofibers Using Human Mesenchymal Stem Cells

**Figure 4:**
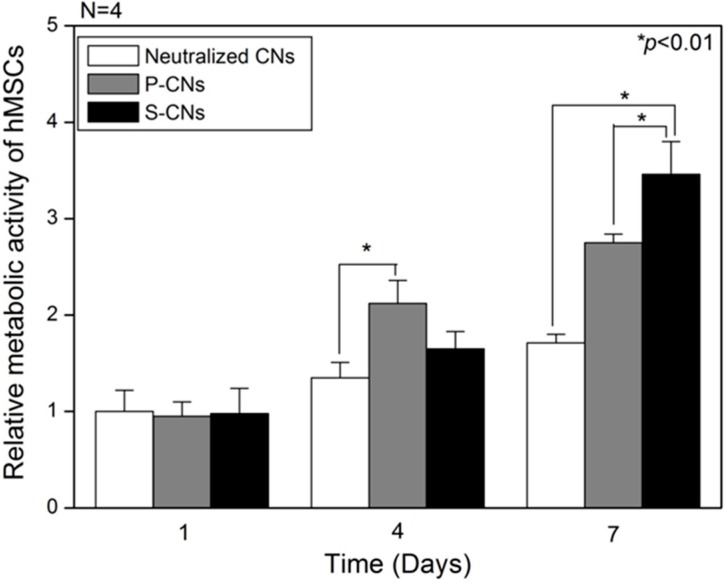
The relative metabolic activity of hMSCs on neutralized CNs, P-CNs and S-CNs in conditioned (serum free) media over the course of seven days.

Next, we evaluated the metabolic activities of hMSCs on CNs using the Cell Counting Kit-8 (CCK-8, Dojindo Laboratories, Kumamoto, Japan) assay. As shown in Figure 4, on Day 1 of culture, the metabolic activities of hMSCs were not significantly different among neutralized CN (1.0 ± 0.22), P-CN (0.95 ± 0.15) and S-CN (0.98 ± 0.26) samples. However, on Day 4, the metabolic activities of hMSCs on P-CN samples rapidly increased to 2.12 ± 0.24, compared to the slight increase for neutralized samples (1.35 ± 0.16) and SP-incorporated samples (1.65 ± 0.18). This enhanced induction of hMSC proliferation could be explained by the greater release of passively-adsorbed SP compared to other groups. On Day 7, the metabolic activities of hMSCs on SP-incorporated samples, S-CNs (3.46 ± 0.34), was higher than that for neutralized CNs (1.71 ± 0.09) and P-CNs (2.75 ± 0.08). The growth of hMSCs cultured on SP-incorporated substrates was greater than that of other groups up to and after seven days in culture. Thus, these results suggest that locally high concentrations and confined release of SP could account for the enhanced cellular activities of hMSCs. As consistent results, Hong *et al*. reported that SP enhanced the growth of MSCs, even though the SP (at a similar concentration) was physically adsorbed or covalently bound [10].

**Figure 5:**
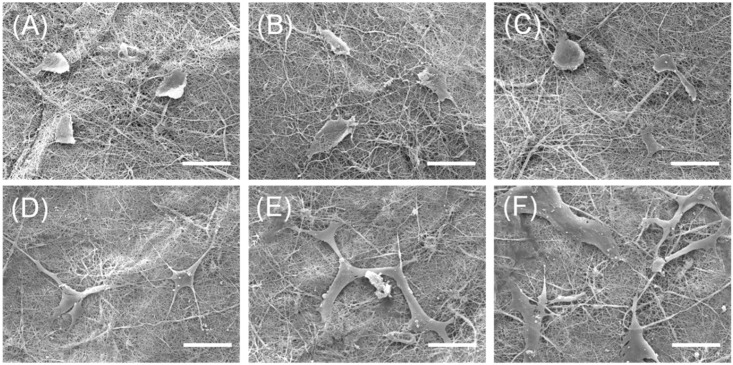
Representative SEM images of hMSCs cultured on neutralized CNs, P-CNs and S-CNs for seven days. (**A**–**C**) SEM images of hMSCs on neutralized CNs (**A**), P-CNs (**B**) and S-CNs (**C**) on Day 1; (**D**–**F**) hMSCs on neutralized CNs (**D**), P-CNs (**E**) and S-CNs (**F**) on Day 7. Scale bar: 50 μm.

Then, the effects of SP on cultured hMSCs were confirmed by SEM analyses. On Day 1, the cultured hMSCs on all CNs samples exhibited spindle- or round-shaped cell morphologies, which were not significantly different regardless of the presence of SP, as shown in Figure 5. However, after seven days, hMSCs on SP-incorporated substrates (S-CNs) showed a larger number of cell density, compared to those of other substrates, showing a mature filopodia (Figure 5F). That is, hMSCs on neutralized substrates showed thinner morphologies and lower cells density, compared to hMSCs on SP-incorporated substrates. These morphological differences of hMSCs on nanofibers were correlated with CCK-8 assays (Figure 4).

**Figure 6:**
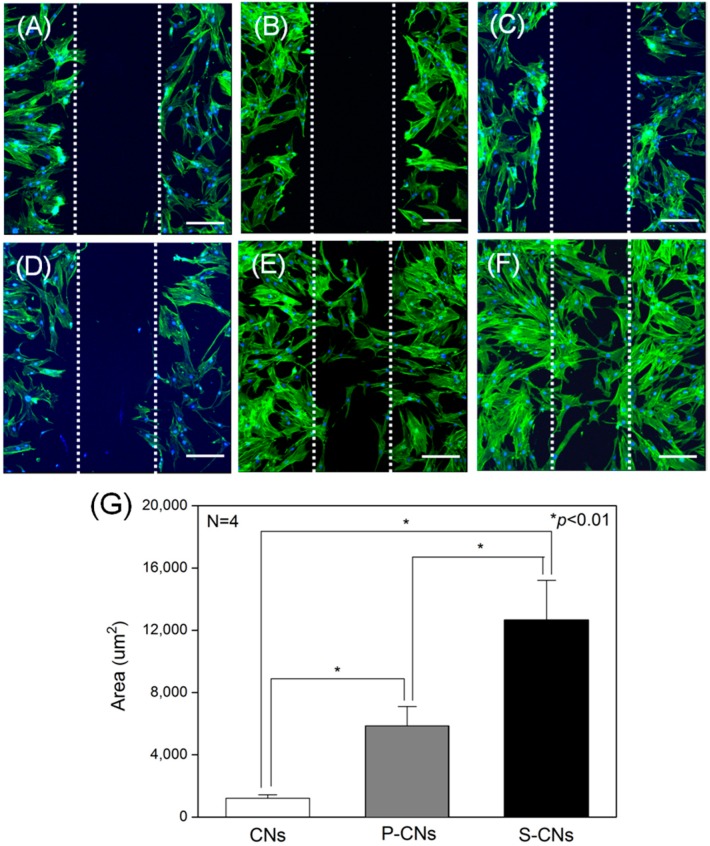
hMSCs were cultured on CNs in an *in vitro* wound-healing model. (**A**–**C**) Dotted white lines represent initial wound edges at 0 h for neutralized CNs (**A**), P-CNs (**B**) and S-CNs (**C**); (**D**–**F**) After 48 h, cells on neutralized CNs (**D**), P-CNs (**E**) and S-CNs (**F**) were stained for actin filaments (green) and nuclei (blue) using FITC-phalloidin and DAPI, respectively. Scale bar: 50 μm; (**G**) Quantitative analysis of the migrated area of hMSCs from (**D**–**F**).

Next, we evaluated the effects of immobilized SP on the migration behavior of hMSCs seeded on CNs, as shown in Figure 6. An initial wound edge of approximately 100 μm was prepared across the length of each sample using sterile syringe needles (Figure 6A–C). After 48 h in culture, hMSCs cultured on P-CNs and S-CNs (Figure 6E,F) migrated faster and in larger numbers compared to the rare migration of hMSCs cultured on neutralized CNs, an effect that was dependent on SP concentration (Figure 6D). To quantify this migratory behavior, we evaluated the migrated area from fluorescence images. As shown in Figure 6G, the migrated area of hMSCs on neutralized CNs was 1209.83 ± 226.48 μm^2^. In keeping with the results shown in Figure 6E,F, the area of hMSCs increased to 5854.48 ± 1246.57 and 12,763.69 ± 2537.54 μm^2^ for the P-CN and S-CN samples, respectively. These migration behaviors of hMSCs are consistent with previous reports that SP plays a strong chemotaxis role, inducing hMSC migration into the periphery *in vitro* and *in vivo* [11,19,20]. Notably, the biological activity of SP was preserved in SP-immobilized nanofibers, a possible critical factor for *in vivo* applications.

### 2.4. In Vivo Evaluation of Foreign Body Reaction and Recruited Stem Cells

**Figure 7:**
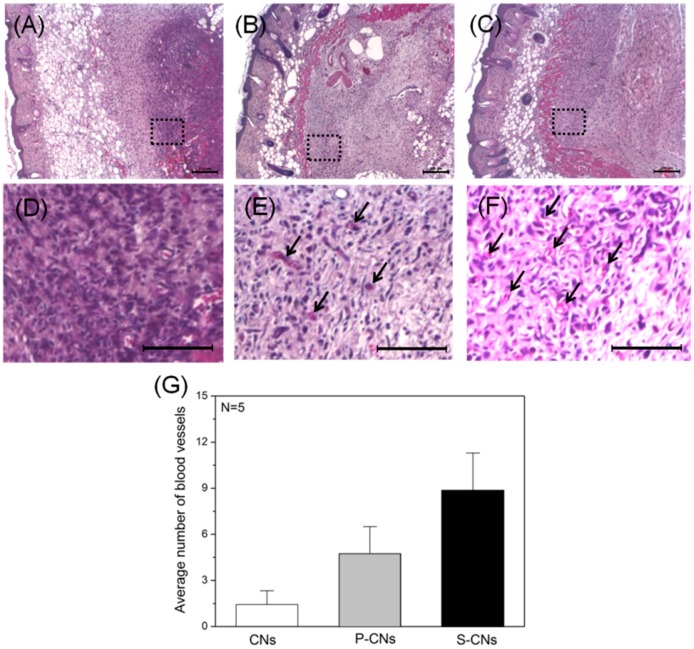
Histological images of implanted CNs H&E-stained after one week. (**A**–**C**) Images of neutralized CNs (**A**), P-CNs (**B**) and S-CNs (**C**) samples within subcutaneous tissue; (**D**–**F**) Magnified images of the dotted line regions in (**A**–**C**), respectively. SP-incorporated samples (**B**,**C**) showed weaker foreign body reactions (FBRs) and larger numbers of blood vessels (dark arrows) compared to other samples. Scale bar: 200 μm; (**G**) Quantitative analysis of blood vessels from (**D**–**F**).

To evaluate the *in vivo* biological effects of neutralized and SP-incorporated CNs, we subcutaneously implanted each sample into Balb/C nude mice. H&E-stained images of each sample one week after implantation (Figure 7A–D) show that neutralized CNs evoked an FBR that was mediated by inflammatory host cells (stained with dark violet). However, SP-incorporated CNs showed a weaker FBR and a larger number of blood vessels (Figure 7E,F, black arrows) around implants.

**Figure 8:**
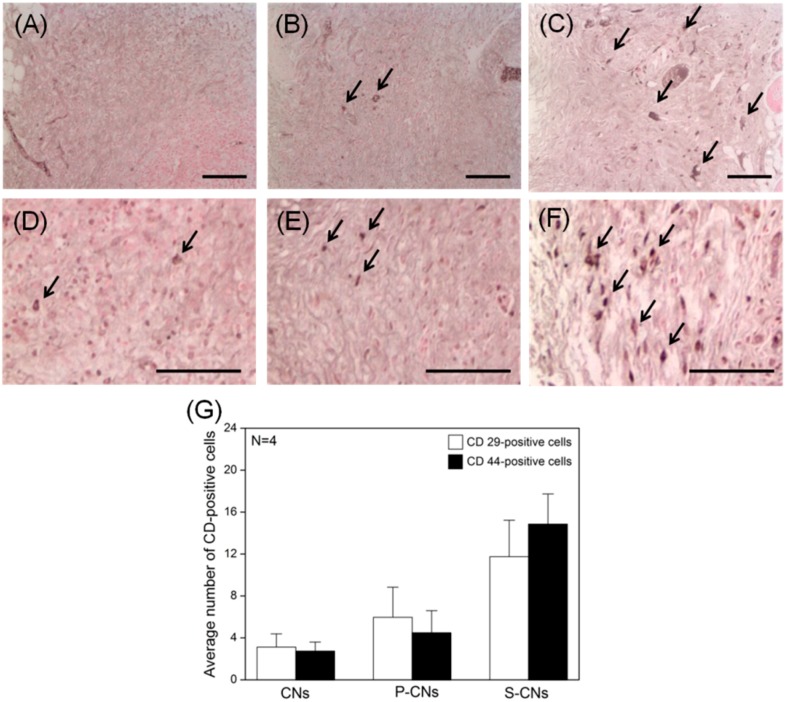
*In vivo* hMSC recruitment in CNs retrieved after one week. (**A**–**C**) Images showing immunohistochemical staining of CD29-positive hMSCs (dark arrows) in neutralized CNs (**A**), P-CNs (**B**) and S-CNs (**C**); (**D**–**F**) Images of CD44-positive hMSCs (dark arrows) in neutralized CNs (**D**), P-CNs (**E**) and S-CNs (**F**). SP-incorporated samples showed a larger number of CD29- and CD44-positive cells compared to the pristine sample. Scale bar: 100 μm. (**G**) Quantitative analysis of CD marker-positive hMSCs from (**A**–**F**).

Finally, to evaluate the response of host stem cells to CNs, we performed immune-histochemical staining of each explanted CN for the hMSC-specific markers CD29 and CD44, as well as integrin β-1 and glycoprotein, at seven days post-implantation. As shown in Figure 8, these *in vivo* experiments revealed that the proportion of CD29- and CD44-positive hMSCs was significantly lower in neutralized CNs than in SP-conjugated groups (Figure 8A–D). A quantitative analysis of recruited hMSCs, performed by evaluating the proportion of CD marker-positive cells from immunohistochemically-stained images (Figure 8G), showed that the average number of CD29- and CD44-positive hMSCs was 3.12 ± 1.27 and 2.75 ± 0.86, respectively. The number of recruited CD29-positive hMSCs for P-CNs and S-CNs increased to 5.97 ± 2.87 and 4.46 ± 2.14, respectively, whereas the corresponding increases for CD44-positive cells were 11.75 ± 3.47 and 14.86 ± 2.86. Based on our *in vitro* SP-release studies, these results suggest that SP covalently conjugated to CNs maintains its biological activity, is released slowly and is capable of recruiting MSCs *in vivo* [17]. More importantly, the strategy of using bioactive molecules may allow stem cell recruiting factors to persist longer in an implant, allowing them to promote long-term tissue regeneration [21]. Although the specific source of the recruited hMSCs is not yet known, bone marrow and/or adipose tissues are possible origins [22].

Our study showed that SP-incorporated CNs promote the cellular responses of hMSCs *in vitro* and *in vivo*, compared to neutralized CNs. Notably, SP incorporated on a matrix, maintained its biological activity and could greatly enhance the recruitment of hMSCs into implanted nanofibers under *in vivo* conditions. Moreover, various combinations of regulatory bioactive molecules, such as cytokines and cell adhesion molecules, could be immobilized to the positive amino groups of chitosan to simultaneously enhance local induction, migration and recruitment of MSCs. Taken together, these findings suggest that our SP delivering system could be extended to various therapeutic applications using MSC for the repair of tissue injury.

## 3. Experimental Section

### 3.1. Materials

Chitosan (*M*_W_: 370 kDa; deacetylation degree: 75%), dichloromethane (MC), trifluoroacetic acid (TFA), dimethyl sulfoxide (DMSO), 4-morpholine ethane sulfonic acid sodium salt (MES), sodium hydroxide (NaOH), bovine serum albumin (BSA), Triton X-100, paraformaldehyde and hexamethyldisilazane (HMDS) were obtained from Sigma-Aldrich (St. Louis, MO, USA). Methanol and ethanol (absolute grade) were purchased from Merck (Darmstadt, Germany). Low-glucose Dulbecco’s Modified Eagle’s Medium (DMEM) and 0.05% trypsin-EDTA were purchased from Gibco BRL (Carlsbad, CA, USA). Dulbecco’s phosphate-buffered saline (DPBS), penicillin-streptomycin (p/s) and fetal bovine serum (FBS) were purchased from WelGene Inc. (Daegu, Korea). SP (*M*_W_: 1347.6 Da) was purchased from Calbiochem (Frankfurter, Darmstadt, Germany).

### 3.2. Fabrication of Chitosan Nanofibers

Following an electrospinning process, chitosan solution (5 wt % in a TFA:MC = 7:3 volume ratio) was injected into a glass syringe with a stainless needle using a syringe pump (KD Scientific, Holliston, MA, USA) at a 1 mL/h flow rate. Eighteen kilovolt voltages were applied between the positive-charged needle and the negative-charged collector by a power supply (Wookyung Tech, Incheon, Korea). The collecting chitosan nanofibers were dried at room temperature overnight. Then, a neutralizing process was conducted to remove acidic components using a NaOH (3 M dissolved in absolute methanol) for 0.5 h [18]. After the neutralizing process, nanofibers were washed with methanol solution (70% and 50% diluted in deionized water (DI)), sequentially. Finally, neutralized samples were freeze-dried for 24 h.

### 3.3. SP Coupling Process on Chitosan Nanofibers

The SP coupling process on CNs was conducted by the covalent conjugation technique [17]. Briefly, the neutralized samples were immersed and agitated in MES buffer (0.1 M pH 6.7) containing *N*-hydroxysuccinimide (NHS, 0.1 mM) at room temperature for 2 h. After the washing process using MES buffer, chitosan samples were immersed in MES buffer contained the designed concentration (0, 10, 50, 100 and 500 ng per 1 mg sample) of SP and then were agitated for 10 h to designate SP-conjugated chitosan nanofibers (S-CNs). As the control group, P-CNs samples were prepared using passively-adsorbed SP of 100 ng on nanofibers. During the washing and freeze-drying process, we obtained CNs, P-CNs and S-CNs samples.

### 3.4. Morphological Analysis

The morphology of nanofibers was observed using a MIRA II field emission-scanning electron microscope (FE-SEM; Tescan, Brno, Czech Republic). Before analysis, samples were coated with gold using an electron beam coater (Eiko IB3; Tokyo, Japan). The average diameter of fiber was measured using ImageJ (National Institutes of Health, Bethesda, MD, USA) from each of the 10 selected field’s image.

### 3.5. Quantification of SP on Nanofibers

The amount of SP on nanofibers was quantified using an enzyme-linked immune sorbent assay (ELISA) kit (R&D Systems, McKinley, MN, USA). A 50-μL solution contained SP, which remained in solution after the SP coupling process, was mixed with 50 μL diluent buffers. Then, the mixed solution was incubated in an ELISA plate for 2 h. After the rinsing process, 100 μL of biotin conjugate and streptavidin were added to the ELISA plate sequentially. After incubation for 30 min, stop solution was added to the ELISA plates. Finally, the optical density (OD) of the solution in each well was read using a micro plate reader (Spectra Max M2e, Molecular Devices, Sunnyvale, CA, USA). A standard calibration curve was determined using known concentrations of SP solutions.

### 3.6. Metabolic Activities of hMSCs on Nanofibers

hMSCs (Lonza, Basel, Switzerland) were seeded onto the CNs at a density of 2000 cells/cm^2^ (Passage Number 7) in DMEM containing 10% FBS and 1% p/s. After 1 day of culture, the culturing medium was changed to conditioned medium (DMEM containing 1% FBS and 1% p/s). The metabolic activities of hMSCs on nanofibers were evaluated at 1, 4 and 7 days using CCK-8 assays. Briefly, cells cultured on samples (circular shape, diameter = 15 mm, *n* = 4) were washed with PBS, and 500 μL CCK solution were reacted with the cell cultured samples. After incubation of 3 h, the OD value was measured at 450 nm using a micro plate reader.

For morphological analysis of hMSCs, each sample (at 1 and 7 days) was fixed in 3.7% paraformaldehyde for 3 h. Then, the CNs with cells were dehydrated with increasing concentrations of ethanol (30%, 50%, 70%, 90% and 100%) for 10 min each. Then, the nanofibers with cells were immersed with HMDS to further water elimination. The morphology of hMSCs on CNs was investigated using an FE-SEM.

### 3.7. Migration of hMSCs on Nanofibers

To examine cell migration behavior, hMSCs were seeded as monolayers on CNs at a density of 2 × 10^4^ cells/cm^2^, as described above (Section 3.6), and a gap defect or “artificial wound” was created across the length of the nanofibers by sterile syringe needles. Then, hMSCs were allowed to migrate into the gap defect for 48 h. After cultivation, hMSCs were stained with 4′,6-diamidino-2-phenylinodole (DAPI, Invitrogen, Waltham, MA, USA) and Alexa Fluor 488 phalloidin (Invitrogen) to determine the initial wound edges and to monitor the wound coverage. For quantitative analysis, the area of migrated hMSCs on the nanofibers (*n* = 4) was measured using ImageJ software (National Institutes of Health, Bethesda, MD, USA).

### 3.8. In Vivo Evaluation of Implanted Nanofibers

All animals were treated in accordance with the Institutional Animal Care and Use Committee (IACUC) at Korea Institute of Radiological and Medical Sciences, Republic of Korea. Female, nude mice (Balb/C, 6 weeks, 20–25 g, *n* = 4) were obtained from Narabio tech (Seoul, Korea). EO gas-sterilized CNs (circular shape, diameter = 6 mm) were implanted in incised subcutaneous pockets (10 mm × 10 mm), and then, these were sutured using silk sutures. After 7 days, samples were excised and fixed in 3.7% paraformaldehyde, embed in paraffin and sliced into 10-µm sections (obtaining five slide sections per animal) using a microtome. For evaluation of FBR, the resulting samples were stained with H&E (hematoxylin and eosin, Sigma-Aldrich, St. Louis, MO, USA). Furthermore, to evaluate the stem cell recruitment, samples were immunohistochemically stained with CD29 (rabbit monoclonal anti-integrin β 1 antibody EP1041Y, 1:1000, Abcam, Cambridge, UK) and CD44 (rabbit monoclonal antibody EPR1013Y, 1:1000, Abcam) diluted with normal horse serum buffers (Vector Laboratories, Burlingame, CA, USA) for 1 h. Additionally, the samples were washed three times with DPBS and stained with biotin-conjugated secondary antibodies. After 1 h incubation, samples were washed three times with DPBS and incubated with VECTASTAIN ABC reagents (Vector Laboratories). Then, samples were incubated with streptavidin peroxidase substrate solution and DAB (Vector Laboratories) for 30 min sequentially and rinsed two times with PBS. For the quantitative analysis, each of the five selected fields (0.05 mm^2^, central and four corner regions per slide) was photographed using an optical microscope (BX50, Olympus, Shinjuku, Tokyo, Japan) at a 200× magnification. The number of blood vessels and CD marker-positive cells were measured using ImageJ software from H&E and immunohistochemically-stained images, respectively.

### 3.9. Statistics

Quantitative data were calculated in triplicate and were presented as mean values with standard deviations. Statistical analysis was conducted using ANOVA, followed by Tukey’s HSD test for multiple comparisons. *p* < 0.05 was considered statistically significant.

## 4. Conclusions

In our study, SP-incorporated chitosan nanofibers were successively prepared via a peptide-coupling process. The SP-incorporated nanofibers exhibited morphological stability as a culturing substrate for hMSCs and were able to control the amount of immobilized SP. During *in vitro* studies, SP-incorporated chitosan substrates induced the enhanced cellular metabolic activity and migration of hMSCs compared to pristine samples. Moreover, we observed recruitment of MSCs into SP-incorporated nanofibers *in vivo*. Finally, our SP-delivering systems hold promising potential for controlling biological functions in regenerative medicine.
